# Urinary Mercapturic Acids to Assess Exposure to Benzene and Other Volatile Organic Compounds in Coke Oven Workers

**DOI:** 10.3390/ijerph17051801

**Published:** 2020-03-10

**Authors:** Gianfranco Frigerio, Laura Campo, Rosa Mercadante, Danuta Mielżyńska-Švach, Sofia Pavanello, Silvia Fustinoni

**Affiliations:** 1 Department of Clinical Sciences and Community Health, Università degli Studi di Milano, 20122 Milan, Italy; 2 Environmental and Industrial Toxicology Unit, Fondazione IRCCS Ca’ Granda Ospedale Maggiore Policlinico, 20122 Milan, Italy; 3 Department of Medical Biology and Genetics, Faculty of Medicine, WST University of Technology, 40-555 Katowice, Poland; 4 Department of Cardiac, Thoracic, Vascular Sciences and Public Health, University of Padova, 35128 Padua, Italy

**Keywords:** volatile organic compounds, mercapturic acids, coke oven workers, steel industry workers, S-phenyl mercapturic acid, human biomonitoring

## Abstract

Coke production was classified as carcinogenic to humans by the International Agency for Research on Cancer. Besides polycyclic aromatic hydrocarbons, coke oven workers may be exposed to benzene and other volatile organic compounds (VOCs). The aim of this study was to assess the exposure to several VOCs in 49 coke oven workers and 49 individuals living in the same area by determining urinary mercapturic acids. Active tobacco smoking was an exclusion criterion for both groups. Mercapturic acids were investigated by a validated isotopic dilution LC-MS/MS method. Linear models were built to correct for different confounding variables. Urinary levels of N-acetyl-S-phenyl-L-cysteine (SPMA) (metabolite of benzene), N-acetyl-S-(2-hydroxy-1/2-phenylethyl)-L-cysteine (PHEMA) (metabolite of styrene), N-acetyl-S-(2-cyanoethyl)-L-cysteine (CEMA) (metabolite of acrylonitrile), N-acetyl-S-[1-(hydroxymethyl)-2-propen-1-yl)-L-cysteine and N-acetyl-S-(2-hydroxy-3-buten-1-yl)-L-cysteine (MHBMA) (metabolites of 1,3-butadiene) were 2–10 fold higher in workers than in controls (*p* < 0.05). For SPMA, in particular, median levels were 0.02 and 0.31 µg/g creatinine in workers and controls, respectively. Among workers, coke makers were more exposed to PHEMA and SPMA than foremen and engine operators. The comparison with biological limit values shows that the exposure of workers was within 20% of the limit values for all biomarkers, moreover three subjects exceeded the restrictive occupational limit value recently proposed by the European Chemicals Agency (ECHA) for SPMA.

## 1. Introduction

Volatile organic compounds (VOCs) are defined, according to the European Union [1], as any organic compound having an initial boiling point less than or equal to 250 °C measured at a standard atmospheric pressure. Some VOCs are toxic to humans and some of them have also been classified as carcinogenic to humans by the International Agency for Research on Cancer (IARC), including 1,3-butadiene and benzene (known carcinogenic, group 1) [2,3], acrylamide and styrene (probable carcinogenic, group 2A) [4,5], propylene oxide and acrylonitrile (possible carcinogenic, group 2B) [4,6]. Moreover, chronic exposure to VOCs is associated with respiratory, neurological, reproductive, and developmental effects [7,8,9,10]. Exposure to toxic VOCs may derive both from occupational and non-occupational sources [2,11], with tobacco smoking as the most important non-occupational source in smokers [12].

Biomonitoring is a useful approach to assess the exposure to VOCs in human subjects. It consists in the analysis of toxicants, or their specific metabolites, in the subjects’ biological fluids, such as urine. Its main advantage is the potential to assess the body burden of a given toxicant including all sources and exposure routes. Several mercapturic acids are useful biomarkers of exposure to VOCs. Mercapturic acids are N-acetyl cysteine derivatives of electrophilic compounds initially conjugated with glutathione and then biotransformed to highly hydrosoluble chemicals excreted in urine [13,14]. For the sake of occupational risk assessment, biological limit values and general population reference values have been recommended by committees of experts such as the American Conference of Governmental Industrial Hygienists (ACGIH) [15], the MAK-Commission (German Committee for the determination of occupational exposure limits of the Deutsche Forschungsgemeinschaft, German Research Foundation, DFG) [16] and the Risk Assessment Committee of the European Chemicals Agency (ECHA) [17]. In particular, N-acetyl-S-phenyl-L-cysteine (SPMA) has been recommended as a biomarker of exposure to benzene by all these committees. A summary of the biological limit values for the mercapturic acids of VOCs is reported in Table 1.

Coke, a porous fuel with a high carbon content and few impurities, is essential for the manufacture of steel. It is produced by heating coal in the absence of oxygen in a process called destructive distillation, in order to remove volatile components. Coke production has been classified as carcinogenic to humans (group 1 according to IARC classification) [2]. The primary chemical exposure in coke oven workers is to polycyclic aromatic hydrocarbons (PAHs) [2,18,19]. Moreover, coke oven workers might also be exposed to VOCs emitted during destructive distillation. Indeed, a certain number of studies assessed the exposure to VOCs in coke oven workers quantifying their levels in workplace and breathing zone air, with overall levels ranging from 22.6 µg/m^3^ to 2.17 mg/m^3^ [20,21,22,23,24,25,26,27,28]. Only a few studies performed biomonitoring of VOC metabolites in coke oven workers. In particular, the levels of the benzene metabolites trans, trans-muconic acid [28,29,30,31] and S-phenyl mercapturic acid (SPMA) [28,29,30,31,32,33] and the toluene metabolite S-benzyl mercapturic acid (SBMA) [31] have been measured in the urine of coke oven workers. However, the urinary concentrations of these metabolites have been reported to be highly affected by smoking habit: e.g., Lovreglio and co-workers reported median levels of SPMA equal to 1.35 µg/g creatinine in smoking coke oven workers versus 0.23 µg/g in non-smoking workers [28].

The aim of the present study was to assess the exposure to VOCs in coke oven workers, through the determination of seventeen mercapturic acids in the workers’ urine. This was achieved by applying a recently developed and validated isotopic dilution liquid chromatography–tandem mass spectrometry (LC-MS/MS) method [34]. The exposure was then compared with that of a matched group of individuals belonging to the general population; further comparisons were performed with occupational limit values and reference values. To avoid the known confounding effect of cigarette smoking, the study was performed enrolling only non-smokers.

## 2. Materials and Methods 

### 2.1. Study Design

The enrollment of subjects, investigation design, and sample collection have been described in previous works [35,36]. Briefly, the study included 49 coke oven workers and 49 subjects form the general population living in the same area (controls), with mean age 39.3 (20–59) and 39.7 (21–58), respectively. All subjects were males living in Poland. Active tobacco smoking was an exclusion criterion for this enrolment. Informed consent was signed from each participant and the study was approved by the Ethic Committee of the Institute of Occupational Medicine and Environmental Health in Sosnowiec.

A questionnaire was administered to each subject by trained interviewers, including: personal characteristics, residence, industrial and/or heavy traffic exposure in living place, type of heating at and near home, recent food habits, and hobbies at home involving exposure during the last three days. Workers were also asked about the use of protective equipment and whether their skin was dirty at the end of the work-shift. Workers were from three different plants: 17 worked in a plant producing low-phosphor coke and broken coke (plant J), 24 in a plant producing domestic coke (plant D), and 8 in a plant producing foundry and blast furnace coke (plant R). Among all workers, 11 were expert foremen; 13 were engine drivers, operators, or machine workers; while 25 were coke makers or gas workers. Control subjects, living in the same area and matched for age and gender to workers, were enrolled among clerks involved in a health check-up program at the Institute of Occupational Medicine and Environmental Health in Sosnowiec (Poland).

The collection of a urine sample was performed in workers at the end of the work-shift and after at least three consecutive working days, while it was performed in the late afternoon for control subjects. Urine samples were stored at −20 °C. Cotinine concentrations were quantified with a previously published method via LC-MS/MS [37]. Urinary creatinine was measured using the Jaffè colorimetric method [38].

Although active tobacco smoking was an exclusion criterion, the analysis of urinary cotinine showed that three control subjects had cotinine levels greater than 50 µg/L (212, 195, 668 µg/L), which was considered as the cutoff to distinguish active smokers from persons exposed to passive smoke [39]. Furthermore, most of the other subjects (87%) had levels of cotinine greater than the limit of quantitation (LOQ) (0.1 μg/L), with a median value equal to 2.2 µg/L (<LOQ and 12.5 µg/L, 5th and 95th percentile respectively), showing that most subjects were exposed to passive smoking.

### 2.2. Mercapturic Acid Analysis

The analysis of mercapturic acids, were carried out with a validated LC-MS/MS isotopic dilution method [34]. The presence of 17 mercapturic acids was investigated (Table 2), in particular: N-acetyl-S-(2-hydroxypropyl)cysteine (2-HPMA) (metabolite of propylene oxide), N-acetyl-S-(3-hydroxypropyl)cysteine (3-HPMA) (metabolite of acrolein), N-acetyl-S-(carbamoylethyl)-L-cysteine (AAMA) (metabolite of acrylamide), N-acetyl-S-(N-methylcarbamoyl)-L-cysteine (AMCC) (metabolite of N,N-dimethylformamide), N-acetyl-S-(2-cyanoethyl)-L-cysteine (CEMA) (metabolite of acrylonitrile), N-acetyl-S-(3-carboxy-2-propyl)-L-cysteine (CMEMA) (metabolite of crotonaldehyde), N-acetyl-S-(3,4-dihydroxybutyl)-L-cysteine (DHBMA) (metabolite of 1,3-butadiene), N-acetyl-S-ethyl-L-cysteine (EMA) (metabolite of ethylating agents), N-acetyl-S-(2-hydroxy-3-propionamide)-L-cysteine (GAMA) (metabolite of acrylamide), N-acetyl-S-(2-hydroxyethyl)-L-cysteine (HEMA) (metabolite of acrylonitrile and ethylene oxide), N-acetyl-S-(3-hydroxypropyl-1-methyl)-L-cysteine (HMPMA) (metabolite of crotonaldehyde), (R,S)-N-acetyl-S-[1-(hydroxymethyl)-2-propen-1-yl)-L-cysteine + (R,S)-N-acetyl-S-(2-hydroxy-3-buten-1-yl)-L-cysteine (MHBMA) (metabolites of 1,3-butadiene), N-acetyl-S-methyl-L-cysteine (MMA) (metabolite of methylating agents), S-(4-nitrophenyl)mercapturic acid (NANPC) (metabolite of 4-chloronitrobenze), N-acetyl-S-(2-hydroxy-1-phenylethyl)-L-cysteine + N-acetyl-S-(2-hydroxy-2-phenylethyl)-L-cysteine (PHEMA) (metabolites of styrene), N-acetyl-S-benzyl-L-cysteine (SBMA) (metabolite of toluene), and N-acetyl-S-phenyl-L-cysteine (SPMA) (metabolite of benzene).

### 2.3. Data Elaboration and Statistical Analysis

The MultiQuant™ software (version 3.0.8664.0; Ab Sciex S.r.l, Milano, Italy) was used for data integration. Statistical analysis was performed using R (version 3.6.1, R Foundation, Vienna, Austria) [40] with the Rstudio interface (Version 1.2.1335, RStudio Inc., Boston, Massachusetts, United States). The package “tidyverse” was used for data elaboration and visualization [41].

Measurements below the limit of quantitation were replaced with a value equal to half the LOQ before statistical analysis. Values were corrected with creatinine and then descriptive statistics was performed after grouping by workers and controls: in particular, median, 5th, and 95th percentile of the distribution were calculated for each analyte, along with the percentage of samples above LOQ. Data were log_10_ transformed to ensure normal distribution and Student’s t-test was applied to evaluate statistically significant differences in the levels of mercapturic acids between groups.

To assess the determinants of urinary mercapturic acid levels, different multiple linear regression analyses were computed. In each linear model, the dependent variable was the log_10_-transformed concentration of a specific mercapturic acid, while the independent variables were age, urinary creatinine (log_10_ transformed), and urinary cotinine (log_10_ transformed). The other independent variables added one at time and tested as predictors were residence (rural or urban), industrial exposure (no or yes), heavy traffic exposure near home (no or yes), type of heating at home (wood, coal, oil, gas, or other), type of heating near home (wood, coal, oil, gas, or other), heating whole-building (no or yes), individual home heating (no or yes), consumption of grilled/smoked meat during the last 24 hours (no or yes), consumption of grilled/smoked meat (times/week), and hobbies at home involving exposure to mineral oils, tar, soot, combustion fumes from wood, leaves, other combustible materials, and vehicle exhaust fumes during the last 3 days (no or yes). For workers, the following variables were also tested: plant (J, D, or R), job title (foremen, engine operators, or gas workers), use of individual protective equipment (no or yes), use of mask (hours/day), use of gloves (hours/day), use of overalls (hours/day), use of mask on the day before (hours/day), use of gloves in the day before (hours/day), use of overalls on the day before (hours/day), dirty skin in the last 3 days (a little or moderate/a lot), hand dirty in the last 3 days (a little, moderate, or a lot), and face dirty in the last 3 days (a little, moderate, or a lot).

Most of the considered variables were not significantly associated with the considered mercapturic acids and, therefore, not included in the final models. Then, two different linear models were built. Each model was run separately for each mercapturic acid (µg/L), which was imputed as dependent variable using the log_10_-transformed data. The first linear model was built using data from all subjects included in the study (*n* = 94 since four observations were excluded due to missing values) and was aimed to evaluate the differences between workers and controls. The independent variables were: study group (controls or workers), age (years), log_10_ creatinine (g/L), log_10_ cotinine (µg/L), presence of hobbies at home involving exposure during the last 3 days (no or yes). The second linear model was built considering only data from the coke oven workers (*n* = 49), and it was aimed to determine the role played by plant, job title, and dirty skin. The independent variables included in this model were: plant (J = reference, D, or R), job title (foremen = reference, engine operators, or coke markers), dirty skin (no or yes), age (years), log_10_ creatinine (g/L), and log_10_ cotinine (µg/L). 

For all models, regression slopes were exponentiated in order to obtain the geometric mean ratio (GMR).

## 3. Results

The levels of urinary mercapturic acids in the subjects’ urine samples are reported in Table 2, in μg/g creatinine, and in Supplementary Table S1, in μg/L. Concentrations of mercapturic acids were greater than the LOQ in all samples for 2-HPMA, 3-HPMA, AAMA, AMCC, CMEMA, DHBMA, GAMA, and SBMA; quantifiable in most samples (from 86% to 99%) for CEMA, EMA, HEMA, HPMPA, MHBMA, MMA, PHEMA, and SPMA; while NANPC was quantified only in 4% of samples. For this reason, NANPC was not included in statistical analyses.

The results of the Student’s t-test performed to compare controls and workers revealed significant differences for CEMA (*p* < 0.001), MHBMA (*p* = 0.001), PHEMA (*p* < 0.001), and SPMA (*p* < 0.001).

**Figure 1:**
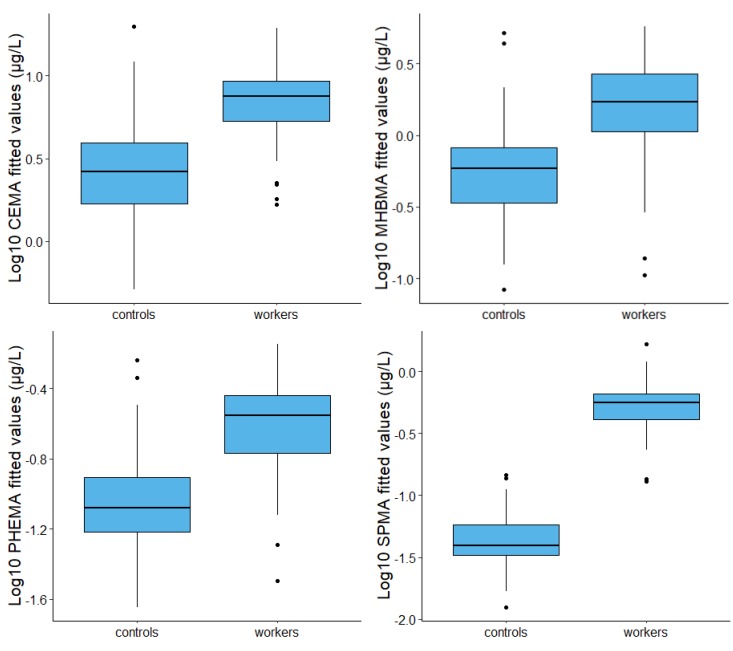
Box plot obtained from fitted values of linear models reported in Table 3, showing the distribution of CEMA, MHBMA, PHEMA, and SPMA in controls and coke oven workers. A box plot is a graphical representation of the data distribution. The box contains the 50% of the observations, with the median dividing the box in two areas and the upper and lower hinge representing the 25th and 75th percentile of the distribution. Outside the box, the upper whisker extends from the hinge to the highest value no further than 1.5 × interquartile range (IQR) from the hinge. The lower whisker extends from the hinge to the smallest value at most 1.5 × IQR of the hinge. Data beyond the whiskers are plotted individually and represented as dots.

The linear model computed to estimate the determinants of each mercapturic acid (µg/L) in workers compared to controls, and corrected for age, creatinine, cotinine, and exposure during last 3 days, showed a significant increase in levels of CEMA (*p* < 0.001, GMR = 1.75), MHBMA (*p* = 0.010, GMR = 2.06), PHEMA, (*p* < 0.001, GMR = 2.15), and SPMA (*p* < 0.001, GMR = 9.53). Furthermore, creatinine was a variable associated with a significant increase of all mercapturic acids (GMR from 6.7 for HEMA to 145.01 for MHBMA); age was associated with a significant increase of AMCC, CMEMA, DHBMA, and SPMA; cotinine was associated with a significant increase of AAMA, AMCC, CEMA, and GAMA; finally, the assessment of activities at home involving exposure during the last 3 days was significantly associated with an increase of AMCC, CEMA, and SPMA, showing a possible non-occupational exposure. The adjusted coefficient of determination (R^2^) ranged from 0.08 (EMA) to 0.56 (SPMA) and was significantly different from zero for all mercapturic acids (Table 3 and Figure 1).

The second linear model built considering only the coke oven workers, which was corrected for age, creatinine, and cotinine, showed that both the production plant and the job title were associated with an increase for some mercapturic acids. In particular, considering the plant J as reference, workers of plant D had significantly higher levels of SPMA (*p* = 0.008, GMR = 3.00), while workers of plant R had significantly higher levels of AAMA (*p* = 0.013, GMR = 2.50). Considering values of foremen as reference, engine operators had no significantly different levels of any of the considered mercapturic acids, while coke makers had significantly higher levels of PHEMA (*p* = 0.023, GMR = 2.04) and SPMA (*p* = 0.032, GMR = 2.71). Workers declaring that their skin was dirty at the end of the work-shift had significantly higher levels of 3-HPMA (*p* < 0.001, GMR = 15.48), DHBMA (*p* = 0.010, GMR = 2.35), GAMA (*p* = 0.029, GMR = 1.78), HMPMA (*p* < 0.001, GMR = 6.70), and SBMA (*p* = 0.018, GMR = 4.13). The adjusted coefficient of determination (R^2^) ranged from 0.05 (HEMA) to 0.56 (GAMA) and was significantly different from zero for all mercapturic acids but for AMCC, CMEMA, EMA, HEMA (Table 4).

## 4. Discussion

In this work, we assessed the occupational exposure to VOCs in coke oven workers using seventeen urinary mercapturic acids as biomarkers.

Higher concentrations of urinary CEMA, MHBMA, PHEMA, and SPMA were found in coke oven workers than in controls, indicating an occupational exposure to acrylonitrile, 1,3-butadiene, styrene, and benzene. However, several other mercapturic acids were similar in these groups, showing that coke oven workers were not exposed to the majority of the considered VOCs (Table 2).

The exposure to VOCs was generally low when compared to occupational limit values. Considering SPMA, metabolite of benzene, the levels in workers were about an order of magnitude lower than the biological exposure indices (BEI) (25 µg/g creatinine) proposed by ACGIH [15]. It is worth mentioning that lower limit values have been recently proposed for benzene. In particular, ECHA has recently proposed a biological limit value (BLV) equal to 2 µg/g creatinine, corresponding to an occupational limit value of 0.2 mg/m^3^ of airborne benzene. Among our study subjects, three workers exceeded this limit. ECHA also suggested a biological guidance value (BGV) equal to 0.5 µg/g creatinine; all subjects in the control group had SPMA levels below than this value, while 15 workers had higher levels [17]. Furthermore, levels of SPMA were comparable with Italian reference values (only non-smokers: 0.18 µg/g creatinine, 95th percentile) [42] and US reference values (3.03 µg/g creatinine, 95th percentile) [43]. Comparing the concentration of the other mercapturic acids with biological reference values (BARs) (Table 1) we note that 3-HPMA (metabolite of acrolein) was higher than the BAR values in seven controls and in five workers (about 12% of the total population in study); AAMA (metabolite of acrylamide) was higher than the BAR values in five controls and two workers (about 7% of the total population in study); DHBMA (metabolite of 1,3-butadiene) was higher than the BAR value in two controls and three workers (about 5% of the total population in study), MHBMA (another metabolite of 1,3-butadiene) was higher than the BAR value in four controls and nine workers (about 13 % of the total population in study). Interestingly, all the subjects (both controls and workers) had levels of AMCC (metabolite of N,N-dimethylformamide) higher than biological tolerance value for occupational exposure (BAT) [16].

The levels of mercapturic acids found in the present study were largely comparable with those previously reported for non-smokers of the general population; differences were noted only for HMPMA and SBMA, with control subjects of this study showing higher levels for HMPMA and lower levels for SBMA than in previous works [44,45,46].

Comparing workers and controls, higher concentrations of CEMA, MHBMA, PHEMA, and SPMA were found in workers, but the increased association with occupational exposure was small in comparison to the association with tobacco smoking. Median levels of CEMA were 3.7 and 1.4 µg/g creatinine in controls and in workers, respectively; while the levels reported in smokers were some orders of magnitude higher with mean/median levels ranging from 72.5 to 240 µg/g creatinine [44,45,47,48]. MHBMA levels were 0.42 and 1.10 µg/g creatinine in controls and in workers, respectively; reported levels of this metabolite for smokers ranged from similar to the levels found in workers (1.08 µg/g creatinine) [44] to an order of magnitude higher (19.5–27.9 µg/g creatinine) [47,48]. PHEMA was significantly higher in workers than in controls with a median level equal to 0.15 versus 0.07 µg/g creatinine. Once again, these results were lower if compared to values reported in smoking subjects, with mean/median values from 0.83 to 2.3 µg/g creatinine [44,48]. SPMA levels were an order of magnitude higher in workers than in controls (median 0.31 vs. 0.02 µg/g creatinine). Nevertheless, levels were still lower if compared with smoking subjects, for which median/mean values close to 1 µg/g creatinine have been reported [44,47].

The possible exposure to benzene in coke oven workers has been reported in previous studies. Dehghani and co-workers determined the levels of benzene, toluene, xylene, and ethylbenzene in the breathing zone air of workers through individual sampling in the coke-making unit of a steel plant and found that benzene levels were higher than the local exposure limits [20]. In another study, toluene and benzene were among the highest VOCs present in the workplace air of the coke-making process; however, the authors evidenced that the concentrations of workplace air pollutants were lower than their local hazardous air pollutant standards for workplace air [25]. In another work, personal air samples were analyzed to determine benzene, toluene, and other VOCs, and it was reported that exposure levels of coke workers were relatively low if compared to the exposure limits [26]. Only a few other studies quantified some mercapturic acids in coke oven workers. In particular, Fan and co-workers determined the concentrations of SPMA and SBMA in coke oven workers and found no significant differences when compared with a control group of farmers. It is noteworthy to mention, though, that the values reported were surprisingly high (median levels of 2.14 and 5.30 µg/g creatinine for SPMA and SBMA, respectively) and this might be due to the presence of tobacco smokers among study subjects, which was not controlled [31]. Lovreglio and co-workers, assessing SPMA levels, revealed that coke oven workers were exposed to a low level of benzene and reported a median level of SPMA in the end-shift urine equal to 0.50 µg/g creatinine (from 0.10 to 6.89 µg/g creatinine); they also reported higher concentrations in oven standpipe workers than in byproduct workers [28]. Previous studies reported urinary levels of SPMA from 3.16 to 34.53 µmol/mol creatinine (6.68–72.96 µg/g creatinine) [32], from 0.4 to 62.6 µmol/mol creatinine (0.85–132.3 µg/g creatinine) [33], from 0.40 to 38.56 µg/g creatinine [29], and from <0.3 to 1020 µg/g creatinine [30].

Urinary levels of mercapturic acids are influenced by several factors, including environmental exposures. The linear models presented in Table 3 aimed to compare workers to controls while correcting for potential confounders. The inclusion of urinary cotinine levels was useful to correct for environmental tobacco smoke: indeed, even though subjects were non-smokers, most of them had quantifiable low levels of cotinine, thus showing an exposure to second-hand tobacco smoke; furthermore, unlike what was declared, three control subjects were probably active smokers due to their very high cotinine levels (>50 µg/L). These linear models highlighted that CEMA, MHBMA, PHEMA, and SPMA were significantly different between controls and workers, with levels about 2-fold (CEMA, MHBMA, and PHEMA) and 10-fold (SPMA) higher in workers, thus showing an exposure to benzene and, with a lower impact, an exposure to acrylonitrile, 1,3-butadiene, and styrene.

Among coke oven workers, the possible determinants of mercapturic acids were studied (Table 4). Some significant differences in companies were found, showing the variability across the different plants: indeed, if compared to the plant producing low-phosphor coke and broken coke (J), significant higher levels of SPMA, metabolite of benzene, were found in the plant producing domestic coke (D), and significant higher levels of AAMA, metabolite of acrylamide, were found in the plant producing foundry and blast furnace coke (R). Comparing job titles, engine operators were similar to foremen, while coke markers had significantly higher concentrations (about 2–3 fold) of PHEMA and SPMA than foremen, thus showing that the task of coke markers was associated with higher exposure to styrene and benzene. The most important determinant of exposure among coke oven workers was the dirty skin variable: indeed, during the interview, workers were asked whether they had dirty skin at the end of the work-shift. The subjects who answered affirmatively had about 15-fold higher levels of 3-HPMA (metabolite of acrolein), 1.5-fold higher levels of DHBMA (metabolite of 1,3-butadiene), 1.8-fold higher levels of GAMA (metabolite of acrylamide), 6.7-fold higher levels of HMPMA (metabolite of crotonaldehyde), and 4-fold higher values of SBMA (metabolite of toluene). Not having the skin dirty could be a sign of higher working skill and/or the correct use of personal protective equipment, and both concur to decrease the body burden of VOCs.

Since it has been extensively reported that active tobacco smoke represents the most important non-occupational exposure to VOCs [12], tobacco smoking was an exclusion criterion during the enrollment. This is a strength of this work, further strengthened by the analysis of urinary cotinine, which revealed the presence of three active smokers and an overall exposure to second-hand smoke. To take this exposure into consideration, urinary cotinine was included as independent variable into the linear regressions. Another strength of this study is that several variables were tested for their inclusion in the linear models, since levels of mercapturic acids might be influenced by several other confounding factors, such as diet [49,50] and environmental pollution [51,52]. Finally, to our knowledge, this is the first time that such a broad spectrum of mercapturic acids has been determined in urine samples of coke oven workers. A limitation of this study is that the results cannot be representative of all workers of a coke plant since, in a coke refinery, other job titles are present such as byproduct workers. Indeed, a previous study assessing occupational exposure through personal air measurements showed that the exposure to benzene, toluene, and xylene was higher in coke byproduct workers, while coke oven workers are more exposed to PAHs [22].

## 5. Conclusions

In conclusion, the quantitation of several urinary mercapturic acids showed that coke oven workers were exposed to higher levels of benzene (about 10-fold), acrylonitrile, 1,3-butadiene, and styrene (about 2-fold) than control subjects. However, the impact of working exposure to the body burden of VOCs was within 20% of existing biological limit values for most biomarkers; for SPMA only three subjects exceeded the restrictive biological limit value recently proposed by the ECHA.

## Figures and Tables

**Table 1:** Biological limit values for mercapturic acids of different volatile organic compounds recommended by the American Conference of Governmental Industrial Hygienists (ACGIH) [15], the Deutsche Forschungsgemeinschaft (DFG) [16], and the European Chemicals Agency (ECHA) [17].

Chemical	CAS Number	Organization	Biomarker	Sampling Time	Biological Value	Value	
Acrolein	107-02-8	DFG	3-HPMA	End of shift/for long-term exposures: at the end of the shift after several shifts	BAR (NS)	600 µg/g creatinine	
Acrylamide	79-06-1	DFG	AAMA	End of shift	BAR (NS)	100 µg/g creatinine	
Benzene	71-43-2	ACGIH	SPMA	End of shift	BEI	25 µg/g creatinine	
		ECHA		End of shift, after several shifts	BLV	2 µg/g creatinine	
					BGV	0.5 µg/g creatinine	
		DFG		End of shift	EKA	Air (mg/m^3^)	Biomarker (µg/g creatinine)
						0.1	1.5 (NS)
						0.2	3 (NS)
						0.5	5
						1.0	12
						2.0	25
						3.3	45
						6.5	90
					BAR (NS)		0.3
1,3-butadiene	106-99-0	DFG	DHBMA	End of shift/for long-term exposures: at the end of the shift after several shifts	EKA	Air (mg/m^3^)	Biomarker (µg/g creatinine)
						0.45	600
						1.1	1000
						2.3	1600
						4.5	2900
						6.8	4200
					BAR (NS)		400
			MHBMA	End of shift/for long-term exposures: at the end of the shift after several shifts	EKA	Air (mg/m^3^)	Biomarker (mg/g creatinine)
						0.45	10
						1.1	20
						2.3	40
						4.5	80
						6.8	120
					BAR (NS)		<2
N,N-dimethylformamide	68-12-2	ACGIH	AMCC	End of shift at end of work week	BEI	30 mg/L	
		DFG			BAT	25 µg/g creatinine	

CAS = Chemical Abstracts Service. NS = non-smokers. BEI = biological exposure indices (ACGIH). EKA = exposure equivalents for carcinogenic substances (DFG). BAR = biological reference value (DFG). BAT = biological tolerance value for occupational exposure (DFG). BLV = biological limit values (ECHA). BGV = biological guidance values (ECHA).

**Table 2:** Median, 5th, and 95th percentile for the levels of mercapturic acids in subjects’ urine samples, expressed as µg/g of creatinine, after grouping by controls and workers. For each compound, the limit of quantitation (LOQ) is also reported, along with the percentage of quantified samples. Finally, the P-value of the Student’s T-test performed on log_10_-transformed values is reported to evaluate differences between the two groups.

Mercapturic Acid	Metabolite of	LOQ (µg/L)	Statistics	Controls (*n* = 49) (µg/g Creatinine)	Workers (*n* = 49) (µg/g Creatinine)	T-Test on Log_10_-Transformed Data (*p*-Value)
2-HPMA	propylene oxide	0.5	Median	3.5	4.8	0.293
			5th–95th	0.9–11.6	1.1–11.1	
			%>LOQ	100	100	
3-HPMA	acrolein	0.2	Median	219.1	215.7	0.092
			5th–95th	81.2–1109.1	26.7–841.1	
			%>LOQ	100	100	
AAMA	acrylamide	3.2	Median	21.3	25.8	0.385
			5th–95th	11.5–117.5	8.9–97.5	
			%>LOQ	100	100	
AMCC	N,N-dimethylformamide	2	Median	112	119	0.272
			5th–95th	40–214	34–256	
			%>LOQ	100	100	
CEMA	acrylonitrile	0.9	Median	1.4	3.7	<0.001
			5th–95th	<LOQ–16.6	1.4–9.6	
			%>LOQ	88	98	
CMEMA	crotonaldehyde	2	Median	300	265	0.613
			5th–95th	99–809	97–1080	
			%>LOQ	100	100	
DHBMA	1,3-butadiene	1.0	Median	177.2	212.7	0.222
			5th–95th	109.7–345.4	96.8–413.5	
			%>LOQ	100	100	
EMA	ethylating agents	0.01	Median	0.04	0.03	0.153
			5th–95th	<LOQ–0.32	<LOQ–0.11	
			%>LOQ	82	90	
GAMA	acrylamide	1.0	Median	5.0	6.0	0.092
			5th–95th	2.5–13.3	3.3–11.8	
			%>LOQ	100	100	
HEMA	acrylonitrileethylene oxide	0.3	Median	0.5	0.6	0.658
			5th–95th	<LOQ–1.6	<LOQ–1.6	
			%>LOQ	86	86	
HMPMA	crotonaldehyde	2	Median	109	101	0.262
			5th–95th	56–270	46–278	
			%>LOQ	100	98	
MHBMA	1,3-butadiene	0.04	Median	0.42	1.10	0.001
			5th–95th	<LOQ–2.47	0.18–3.35	
			%>LOQ	90	96	
MMA	methylating agents	0.09	Median	3.53	2.95	0.175
			5th–95th	0.66–11.28	<LOQ–12.61	
			%>LOQ	100	92	
NANPC	4-chloronitrobenze	0.11	Median	<LOQ	<LOQ	NA
			5th–95th	<LOQ	<LOQ	
			%>LOQ	4	4	
PHEMA	styrene	0.01	Median	0.07	0.15	<0.001
			5th–95th	<LOQ–0.23	0.04–0.4	
			%>LOQ	88	100	
SBMA	toluene	0.02	Median	0.62	0.80	0.316
			5th–95th	0.22–2.00	0.25–3.58	
			%>LOQ	100	100	
SPMA	benzene	0.01	Median	0.02	0.31	<0.001
			5th–95th	<LOQ–0.25	0.04–2.98	
			%>LOQ	71	100	

NA: not assessed.

**Table 3:** Results of the linear models built to evaluate the differences between controls and workers. The dependent variable of each linear model was the log_10_-transformed value of a mercapturic acid (µg/L). The independent variables were study group (controls or workers), log_10_ creatinine (g/L), age (years), log_10_ cotinine (µg/L), hobbies at home involving exposure during the last 3 days (no or yes) (*n* = 94, four observations removed due to missing values).

Mercapturic Acids	Group = Workers (Reference = Controls)	Log_10_ Creatinine (g/L)		Age (years)		Log_10_ Cotinine (µg/L)		Exposure Last 3 Days = Yes (Reference = No)	R^2^ Adj *p*-Value
	GMR (95%CI) *p*-Value	GMR (95%CI) *p*-Value	r (95%CI) *p*-Value	GMR (95%CI) *p*-Value	r (95%CI) *p*-Value	GMR (95%CI) *p*-Value	r (95%CI) *p*-Value	GMR (95%CI) *p*-Value	
2-HPMA2	1.05	20.20	0.54	1.00	−0.01	1.12	0.11	1.36	0.31
	0.75–1.46	7.39–55.17	0.37–0.67	0.98–1.02	−0.21–0.19	0.90–1.39	−0.10–0.31	0.92–1.99	<0.001
	0.77	<0.001	<0.001	0.906	0.904	0.305	0.294	0.117	
3-HPMA3	0.63	26.01	0.43	1.01	0.12	1.19	0.12	1.00	0.16
	0.39–1.02	6.05–111.85	0.25–0.58	0.99–1.04	−0.08–0.32	0.87–1.62	−0.09–0.31	0.57–1.74	<0.001
	0.06	<0.001	<0.001	0.257	0.247	0.28	0.269	0.998	
AAMA	0.98	14.22	0.52	1.01	0.15	1.28	0.26	1.08	0.29
	0.72–1.33	5.62–35.98	0.35–0.65	1.00–1.03	−0.06–0.34	1.05–1.57	0.06–0.44	0.76–1.54	<0.001
	0.889	<0.001	<0.001	0.164	0.154	0.014	0.012	0.677	
AMCC	1.00	10.09	0.56	1.02	0.30	1.20	0.24	1.61	0.39
	0.79–1.27	4.8820.85	0.40–0.68	1.01–1.03	0.11–0.48	1.03–1.40	0.04–0.42	1.22–2.13	<0.001
	0.989	<0.001	<0.001	0.004	0.003	0.022	0.019	<0.001	
CEMA	1.75	7.98	0.41	1.01	0.08	1.88	0.53	1.63	0.52
	1.26–2.42	2.97–21.43	0.22–0.56	0.99–1.02	-0.12–0.28	1.52–2.32	0.37–0.66	1.12–2.38	<0.001
	<0.001	<0.001	<0.001	0.455	0.444	<0.001	<0.001	0.011	
CMEMA	0.95	18.73	0.53	1.02	0.22	0.94	−0.06	0.98	0.25
	0.68–1.31	6.96–50.41	0.37–0.66	1.00–1.03	0.02–0.40	0.76–1.16	−0.26–0.14	0.67–1.43	<0.001
	0.741	<0.001	<0.001	0.039	0.035	0.563	0.555	0.907	
DHBMA	1.05	21.62	0.73	1.01	0.25	1.13	0.2	1.06	0.54
	0.86–1.28	11.77–39.74	0.62–0.81	1.00–1.02	0.05–0.43	0.99–1.29	0.00–0.39	0.84–1.33	<0.001
	0.662	<0.001	<0.001	0.019	0.016	0.061	0.055	0.632	
EMA	0.75	9.02	0.32	1.02	0.14	0.84	−0.12	1.22	0.08
	0.48–1.19	2.23–36.51	0.12–0.49	0.99–1.04	−0.06–0.34	0.63–1.14	−0.31–0.08	0.71–2.07	0.027
	0.226	0.002	0.002	0.176	0.167	0.261	0.25	0.469	
GAMA	1.10	14.62	0.68	1.01	0.16	1.19	0.28	0.99	0.49
	0.90–1.34	7.95–26.88	0.56–0.78	1.00–1.02	−0.05–0.35	1.05–1.36	0.08–0.45	0.78–1.25	<0.001
	0.363	<0.001	<0.001	0.136	0.127	0.009	0.007	0.926	
HEMA	1.00	6.7	0.38	0.99	−0.09	0.98	–0.03	1.33	0.15
	0.73–1.38	2.55–17.63	0.20–0.54	0.98–1.01	−0.29–0.11	0.79–1.20	−0.23–0.18	0.92–1.93	0.001
	0.994	<0.001	<0.001	0.378	0.368	0.814	0.81	0.128	
HMPMA	0.85	13.6	0.49	1	0.01	1.11	0.11	0.99	0.21
	0.62–1.18	5.03–36.79	0.31–0.63	0.98–1.02	−0.19–0.21	0.90–1.38	−0.10–0.30	0.68–1.45	<0.001
	0.341	<0.001	<0.001	0.922	0.921	0.315	0.304	0.96	
MHBMA	2.06	145.01	0.53	1.02	0.14	1.2	0.11	1.01	0.33
	1.19–3.57	27.31–769.98	0.37–0.66	0.99–1.05	−0.06–0.34	0.84–1.72	−0.10–0.30	0.54–1.92	<0.001
	0.010	<0.001	<0.001	0.181	0.171	0.313	0.302	0.966	
MMA	0.63	86.08	0.52	1.01	0.09	0.91	−0.06	1.31	0.24
	0.38–1.05	18.39–402.96	0.36–0.66	0.99–1.04	−0.11–0.29	0.66–1.27	−0.26–0.15	0.73–2.36	<0.001
	0.078	<0.001	<0.001	0.375	0.364	0.591	0.583	0.364	
PHEMA	2.15	36.9	0.54	0.99	−0.09	1.19	0.15	0.99	0.43
	1.46–3.16	11.38–119.66	0.38–0.67	0.97–1.01	−0.29–0.11	0.93–1.53	−0.06–0.34	0.63–1.55	<0.001
	<0.001	<0.001	<0.001	0.38	0.37	0.168	0.159	0.965	
SBMA	1.09	33.41	0.59	1.03	0.33	1.01	0.01	1.37	0.36
	0.78–1.52	12.10–92.27	0.44–0.71	1.01–1.04	0.14–0.50	0.81–1.25	−0.20–0.21	0.93–2.02	<0.001
	0.606	<0.001	<0.001	0.001	0.001	0.962	0.961	0.108	
SPMA	9.53	9.9	0.3	1.01	0.06	1.28	0.15	2.09	0.56
	5.71–15.91	2.09–46.93	0.10–0.47	0.98–1.03	−0.15–0.26	0.91–1.78	−0.05–0.35	1.15–3.79	<0.001
	<0.001	0.004	0.004	0.582	0.574	0.149	0.14	0.016	

**Table 4:** Results of the linear models built to evaluate the differences in levels of considered analytes among workers. The dependent variable of each linear model was the log_10_-transformed value of a mercapturic acid (µg/L). The independent variables were: plant (J = reference, D, or R), job title (foremen = reference, engine operators, or coke markers), dirty skin (no or yes), age (years), log_10_ creatinine (g/L), and log_10_ cotinine (µg/L) (*n* = 49).

Mercapturic Acids	Company = D (Reference = J) GMR (95%CI) *p*-Value	Company = R (Reference = J) GMR (95%CI) *p*-Value	Job Title = Engine Operators (Reference = Foremen) GMR (95%CI) *p*-Value	Job Title = Coke Makers (Reference = Foremen) GMR (95%CI) *p*-Value	Dirty Skin = Yes (Reference = No) GMR (95%CI) *p*-Value	R^2^ Adj *p*-Value
2-HPMA	1.18	1.06	1.29	0.70	0.73	0.23 0.015
	0.71–1.96	0.51–2.19	0.62–2.71	0.39–1.26	0.28–1.90	
	0.504	0.882	0.487	0.230	0.516	
3-HPMA	1.49	1.15	0.86	1.15	15.48	0.31 0.002
	0.67–3.27	0.37–3.57	0.27–2.71	0.46–2.86	3.51–68.30	
	0.317	0.807	0.786	0.755	<0.001	
AAMA	1.04	2.50	1.17	0.85	1.24	0.26 0.007
	0.63–1.70	1.23–5.11	0.57–2.42	0.48–1.50	0.49–3.15	
	0.888	0.013	0.656	0.562	0.646	
AMCC	1.10	1.04	0.79	1.06	2.23	0.15 0.065
	0.69–1.76	0.53–2.04	0.40–1.55	0.62–1.82	0.93–5.35	
	0.673	0.900	0.481	0.821	0.073	
CEMA	1.55	0.91	0.80	1.30	1.09	0.35 <0.001
	0.97–2.46	0.46–1.77	0.40–1.57	0.76–2.22	0.46–2.63	
	0.066	0.772	0.505	0.331	0.836	
CMEMA	1.38	0.87	1.10	1.29	2.67	0.14 0.072
	0.78–2.46	0.38–1.99	0.48–2.56	0.67–2.50	0.90–7.89	
	0.264	0.730	0.815	0.441	0.074	
DHBMA	1.39	1.36	1.21	1.32	2.35	0.50 <0.001
	0.99–1.94	0.84–2.20	0.74–1.98	0.89–1.94	1.24–4.42	
	0.058	0.209	0.443	0.161	0.010	
EMA	1.27	0.61	1.55	1.00	1.32	0.02 0.362
	0.68–2.37	0.25–1.49	0.62–3.88	0.49–2.06	0.41–4.29	
	0.452	0.266	0.336	0.997	0.636	
GAMA	0.88	1.08	1.18	1.27	1.78	0.56 <0.001
	0.67–1.16	0.73–1.60	0.79–1.75	0.93–1.73	1.06–2.96	
	0.362	0.684	0.414	0.134	0.029	
HEMA	0.93	0.60	0.92	1.13	0.62	0.05 0.274
	0.51–1.68	0.25–1.40	0.39–2.18	0.57–2.25	0.20–1.88	
	0.803	0.229	0.841	0.711	0.385	
HMPMA	1.62	2.05	0.99	1.26	6.70	0.34 0.001
	0.94–2.81	0.93–4.51	0.44–2.21	0.67–2.38	2.38–18.81	
	0.082	0.075	0.983	0.461	<0.001	
MHBMA	1.81	1.98	1.04	1.39	2.76	0.23 0.014
	0.88–3.71	0.71–5.57	0.37–2.97	0.61–3.17	0.72–10.65	
	0.103	0.187	0.939	0.428	0.136	
MMA	1.90	1.44	1.07	0.96	2.86	0.22 0.019
	0.74–4.91	0.37–5.65	0.27–4.29	0.32–2.86	0.48–17.03	
	0.179	0.591	0.919	0.940	0.242	
PHEMA	0.71	0.75	1.30	2.04	0.76	0.32 0.002
	0.41–1.20	0.35–1.60	0.60–2.82	1.11–3.77	0.28–2.06	
	0.193	0.444	0.505	0.023	0.577	
SBMA	0.95	0.57	0.83	1.01	4.13	0.18 0.036
	0.51–1.77	0.24–1.40	0.34–2.05	0.49–2.07	1.29–13.29	
	0.873	0.216	0.679	0.976	0.018	
SPMA	3.00	1.21	2.38	2.71	0.92	0.22 0.019
	1.36–6.59	0.39–3.76	0.75–7.50	1.10–6.72	0.21–4.04	
	0.008	0.732	0.136	0.032	0.909	

## Supplementary Materials

The following are available online at https://www.mdpi.com/1660-4601/17/5/1801/s1, Table S1: Median, 5th and 95th percentile for the levels of mercapturic acids in subjects’ urine samples, expressed as µg/L, after grouping by controls and workers. For each compound, the limit of quantitation (LOQ) is also reported, along with the percentage of quantified samples. Finally, *p*-value of the student’s T-test performed on log_10_ transformed values is reported to evaluate differences between the two groups.
