# Supplementary material for: Urinary Mercapturic Acids to Assess Exposure to Benzene and Other Volatile Organic Compounds in Coke Oven Workers
Source: Int J Environ Res Public Health. 2020 Mar 10;17(5):1801. doi: 10.3390/ijerph17051801 (PMC7084241; doi:10.3390/ijerph17051801)
Supplement: Supplementary file 1 [file ijerph-17-01801-s001.pdf]

International Journal of
Environmental Research
and Public Health

MDPI

Urinary Mercapturic Acids to Assess the Exposure to Benzene and Other Volatile Organic Compounds in Coke Oven Workers

Table S1. Median, 5th and 95th percentile for the levels of mercapturic acids in subjects' urine samples, expressed as µg/L, after grouping by controls and workers. For each compound, the limit of quantitation (LOQ) is also reported, along with the percentage of quantified samples. Finally, P-value of the student's T-test performed on log_{10} transformed values is reported to evaluate differences between the two groups.

	LOQ (µg/L)	Statistics	Controls (n=49) (µg/L)	Workers (n=49) (µg/L)	T-test on log_{10} transformed data P-value
**2-HPMA**	0.5	**Median**	5.5	8.9	0.13
		**5th - 95th**	1.4 - 27.2	1.5 - 19.6	
		**%>LOQ**	100	100	
**3-HPMA**	0.2	**Median**	317.6	440.2	0.283
		**5th - 95th**	116.8 - 2109.0	35.1 - 1505.9	
		**%>LOQ**	100	100	
**AAMA**	3.2	**Median**	35.7	54.6	0.151
		**5th - 95th**	13.8 - 212.8	11.8 - 204.8	
		**%>LOQ**	100	100	
**AMCC**	2	**Median**	174	208	0.081
		**5th - 95th**	53 - 445	64 - 520	
		**%>LOQ**	100	100	
**CEMA**	0.9	**Median**	2.3	6.6	<0.001
		**5th - 95th**	<LOQ - 19.0	1.7 - 20.9	
		**%>LOQ**	88	98	
**CMEMA**	2	**Median**	483	531	0.82
		**5th - 95th**	114 - 1744	109 - 2057	
		**%>LOQ**	100	100	
**DHBMA**	1.0	**Median**	307.5	410.4	0.082
		**5th - 95th**	125.3 - 769.2	121.4 - 910.0	
		**%>LOQ**	100	100	
**EMA**	0.01	**Median**	0.06	0.05	0.393
		**5th - 95th**	<LOQ - 0.49	<LOQ - 0.22	
		**%>LOQ**	82	90	
**GAMA**	1.0	**Median**	7.5	11.5	0.032
		**5th - 95th**	3.5 - 27.0	4.5 - 27.7	
		**%>LOQ**	100	100	
**HEMA**	0.3	**Median**	0.9	1.3	0.28
		**5th - 95th**	<LOQ - 2.8	<LOQ - 3.0	
		**%>LOQ**	86	86	
**HMPMA**	2	**Median**	181	205	0.747

		**5th - 95th**	71 - 515	59 - 489	
		**%>LOQ**	100	98	
**MHBMA**	0.04	**Median**	0.85	1.86	0.001
		**5th - 95th**	<LOQ - 6.56	0.29 - 6.67	
		**%>LOQ**	90	96	
**MMA**	0.09	**Median**	6.12	5.89	0.438
		**5th - 95th**	0.59 - 22.07	<LOQ - 21.88	
		**%>LOQ**	100	92	
**NANPC**	0.11	**Median**	<LOQ	<LOQ	NA
		**5th - 95th**	<LOQ - <LOQ	<LOQ - <LOQ	
		**%>LOQ**	4	4	
**PHEMA**	0.01	**Median**	0.11	0.27	<0.001
		**5th - 95th**	<LOQ - 0.41	0.05 - 0.88	
		**%>LOQ**	88	100	
**SBMA**	0.02	**Median**	0.98	1.26	0.141
		**5th - 95th**	0.26 - 4.26	0.32 - 6.21	
		**%>LOQ**	100	100	
**SPMA**	0.01	**Median**	0.03	0.48	<0.001
		**5th - 95th**	<LOQ - 0.33	0.09 - 4.87	
		**%>LOQ**	71	100	

NA: Not assessed
